# Using an Optimization Algorithm to Detect Hidden Waveforms of Signals

**DOI:** 10.3390/s21020588

**Published:** 2021-01-15

**Authors:** Yen-Ching Chang, Chin-Chen Chang

**Affiliations:** 1 Department of Medical Informatics, Chung Shan Medical University, Taichung 40201, Taiwan; nicholas@csmu.edu.tw; 2 Department of Information Engineering and Computer Science, Feng Chia University, Taichung 40724, Taiwan; 3 School of Computer Science and Technology, Hangzhou Dianzi University, Hangzhou 310018, China

**Keywords:** signal detection, hidden waveform, optimization algorithm, discrete Fourier transform, empirical mode decomposition, intrinsic mode function, signal decomposition

## Abstract

Source signals often contain various hidden waveforms, which further provide precious information. Therefore, detecting and capturing these waveforms is very important. For signal decomposition (SD), discrete Fourier transform (DFT) and empirical mode decomposition (EMD) are two main tools. They both can easily decompose any source signal into different components. DFT is based on Cosine functions; EMD is based on a collection of intrinsic mode functions (IMFs). With the help of Cosine functions and IMFs respectively, DFT and EMD can extract additional information from sensed signals. However, due to a considerably finite frequency resolution, EMD easily causes frequency mixing. Although DFT has a larger frequency resolution than EMD, its resolution is also finite. To effectively detect and capture hidden waveforms, we use an optimization algorithm, differential evolution (DE), to decompose. The technique is called SD by DE (SDDE). In contrast, SDDE has an infinite frequency resolution, and hence it has the opportunity to exactly decompose. Our proposed SDDE approach is the first tool of directly applying an optimization algorithm to signal decomposition in which the main components of source signals can be determined. For source signals from four combinations of three periodic waves, our experimental results in the absence of noise show that the proposed SDDE approach can exactly or almost exactly determine their corresponding separate components. Even in the presence of white noise, our proposed SDDE approach is still able to determine the main components. However, DFT usually generates spurious main components; EMD cannot decompose well and is easily affected by white noise. According to the superior experimental performance, our proposed SDDE approach can be widely used in the future to explore various signals for more valuable information.

## 1. Introduction

Signal decomposition (SD) is a very useful tool for analyzing the components of source signals in order to determine interesting and meaningful hidden signal patterns. When analyzing a signal, we usually approximate the signal or function by “atoms,” which may be sinusoids, wavelets, or Gabor functions [1]. In order to find these qualified “atoms” to represent the function, an effective algorithm must be adopted. In general, we choose some “atoms” from a larger collection of “atoms” called a “dictionary” [1]. “Atoms” are signal components—especially, meaningful signal components—and the “dictionary” is a collection of signal components.

In general, source signals contain some precious information in hidden waveforms, and therefore SD plays a very important role in signal analysis. Unlike traditional analysis tools, such as Fourier transform (FT) and series [2], as well as wavelet transform (WT) [1,3], empirical mode decomposition (EMD) [4,5,6] has attracted a great deal of attention over the past two decades as it is a data-driven decomposition tool without preset basis functions.

Huang et al. [4] first reviewed non-stationary data processing methods, such as FT and WT, then discussed their potential problems, and finally proposed their outstanding EMD and Hilbert spectra. In the work by Huang and Shen [6], many related theories and applications were provided. In addition, various fields have also shown the practical value of EMD, including in medicine [7,8], hydrology [9], mechanics [10,11], civil engineering [12] and theoretical analysis [13].

EMD can directly, adaptively and quickly decompose a source signal into a collection of intrinsic mode functions (IMFs) [4,6]; each IMF must satisfy two conditions: (1) in the whole data set, the number of extrema and the number of zero crossings must either equal or differ at most by one; and (2) at any point, the mean value of the envelope defined by the local maxima and the envelope defined by the local minima is zero. Therefore, each IMF can also be viewed as special basis functions; they are directly derived from the data rather than being predetermined by basis functions such as FT and WT.

IMFs are derived through the following sifting process. First, the extrema of a signal are identified; second, all the local maxima are connected by a cubic spline line as the upper envelope and all the local minima are connected by a cubic spline line as the lower envelope; finally, their mean is calculated, and the difference between the data and the mean is the first component. Repeat the procedure until two conditions of IMFs are satisfied. 

IMFs are in turn generated from highest-frequency to lowest-frequency components and finally to the residue. Indeed, these data-derived IMFs sometimes contain some meaningful and valuable signal patterns, but some components are often hard to explain. Therefore, they generally need some auxiliary tools, such as time–frequency or time–frequency–energy distribution, for clarification. 

Furthermore, EMD-related methods are prone to mode mixing [14,15,16], end effects [14,15] and detrend uncertainty [15]. Therefore, a new noise-assisted data analysis tool, called ensemble empirical mode decomposition (EEMD) [14], was proposed to reduce mode mixing and end effects. EEMD consists of one part different from EMD: it sifts an ensemble of white noise-added signal (data), and then treats the mean as the final true result. The aim of finite amplitude white noise is to force the ensemble to exhaust all possible solutions in the sifting process. 

Further, compact empirical mode decomposition (CMED) [15] was proposed to reduce mode mixing, end effects and detrend uncertainty. CMED is composed mainly of two parts: one is to use highest-frequency sampling to generate pseudo extrema for effectively identifying upper and lower envelopes; the other is to use a set of 2*N* (for *N* data points) algebraic equations for determining the maximum (minimum) envelope at each decomposition step.

Zhang et al. [16], with the help of an improved genetic algorithm (GA), also proposed an improved ensemble empirical mode decomposition method (GAEEMD) to solve mode mixing. In the improved GA, Zhang et al. used a difference selection operator instead of a traditional selection operator (roulette selection or tournament selection) and selected the amplitudes of the added white noise and the number of trials as the parameters of their fitness function, which was the reciprocal of an orthogonal index concerning the decomposed IMFs. More exactly, GAEEMD applies an improved GA in the IMFs obtained from the sifting process of EEMD, rather than directly in the source signal; in other words, it indirectly applies an improved GA in the source signal.

For a wide range of applications, EMD has been further extended for use in two-variant (bivariate) [17], three-variant (trivariate) [18] and multiple-variant (multivariate) [19] signals. These methods can extract two-dimensional to multiple-dimensional common oscillatory modes and facilitate the fusion of information from two or multiple sources.

Different from EMD-like methods, Singh et al. [20] also proposed an adaptive decomposition method, which was based on Fourier theory, called the Fourier decomposition method (FDM); it can decompose any data into a small number of Fourier intrinsic band functions (FIBFs) and thus can be viewed as a generalized Fourier expansion with variable amplitudes and frequencies.

Since EMD and FDM act essentially as a dyadic filter bank [21,22,23], some waves within the same dyadic filter bank cannot be decomposed into separate waves, even simple periodic waves. To solve this problem, we proposed a direct decomposition tool to decompose the source signals into separate components.

Similar to GAEEMD, our proposed method also applies another nature-inspired optimization algorithm to search the optimal parameters; in contrast to GAEEMD, we adopted a standard differential evolution (DE) algorithm, which is a real-coded algorithm, rather than a traditional GA, which is a binary-coded algorithm. In addition, we directly selected the amplitudes, frequencies and phases of the source signal as the parameters of the fitness function, which is the mean squared error (MSE) between the source signal and its searched signal.

At a pioneering stage, we selected four combinations of three periodic waves as source signals, including sinusoidal, square and triangular waves. They were a combination of three sinusoidal waves (continuous and smooth), a combination of three square waves (non-continuous and also non-smooth), a combination of triangular waves (continuous but non-smooth) and a composite of the three above-mentioned waves (non-continuous and non-smooth).

In the absence of noise, experimental results show that our proposed SD by DE (SDDE) can perfectly or almost perfectly decompose these four source signals into corresponding separate waves, where their corresponding amplitudes, frequencies and phases are also exactly or almost exactly obtained. In contrast, EMD, limited by a considerably finite frequency resolution, tends to view these simple periodic signal combinations as special basis functions, and it therefore cannot further decompose them into separate components. Similarly, limited by a finite frequency resolution, discrete Fourier transform (DFT) usually generates spurious main components.

Even in the presence of white noise, our proposed SDDE approach can still determine the main signal components; however, EMD is easily affected by white noise, and it therefore cannot decompose contaminated signals into separate components. Likewise, DFT easily generates more spurious main components.

The rest of the paper is organized as follows. Section 2 outlines some related tools for SD and DE. Section 3 presents the problem formulation of the paper. Section 4 gives experimental results and a detailed discussion. Finally, Section 5 concludes with some observations and possible developments for the future.

## 2. Related Tools

In this section, the basics of SD and the standard procedure of DE will be mentioned; some tools of SD include the Fourier series and transform, wavelet transform and EMD.

In the case of SD, a signal $x\left(t\right)$ or function $f\left(t\right)$ can often be analyzed, described, or processed by a linear decomposition [3]

(1) $$f\left(t\right)=\sum_{l}a_{l}\psi_{l}\left(t\right),$$

where l is an integer index for a finite or an infinite sum; $a_{l}$ represents the real-valued expansion coefficients; and $\psi_{l}\left(t\right)$ is a set of real-valued functions of t, called the expansion set. If Equation (1) is unique, the set is called a basis for the class of functions that can be expressed in that way. If the basis is orthogonal, meaning:

(2) $$\langle \psi_{k}\left(t\right),\;\psi_{l}\left(t\right)\rangle =\int \psi_{k}\left(t\right)\psi_{l}\left(t\right)dt=0,\;k\neq l,$$

then the coefficients can be calculated by the inner product:

(3) $$a_{k}=\langle f\left(t\right),\;\psi_{k}\left(t\right)\rangle =\int f\left(t\right)\psi_{k}\left(t\right)dt.$$

For the Fourier series [2], the orthogonal basis functions $\psi_{k}\left(t\right)$ are $\sin\left(k\omega_{0}t\right)$ and $\cos\left(k\omega_{0}t\right)$ with frequencies of $k\omega_{0}$. These are especially suitable for periodic, time-invariant or stationary signals. When the period approaches infinity, the Fourier series tends to the Fourier transform [2].

For the wavelet expansion, a two-parameter system is constructed so that Equation (1) becomes:

(4) $$f\left(t\right)=\sum_{k}\sum_{j}a_{j,k}\psi_{j,k}\left(t\right),$$

where both j and k are integer indices and $\psi_{j,k}\left(t\right)$ represents the wavelet expansion functions that usually form an orthogonal basis.

The set of expansion coefficients aj,k are called the discrete wavelet transform (DWT) of $f\left(t\right)$, and Equation (4) is its inverse transform.

Wavelet transforms are especially suitable for transient, nonstationary or time-varying signals; for applications with nonlinear and non-stationary signals, empirical mode decomposition [4,5,6] is more suitable. The method is data-driven without any basis functions presumed.

Through the sifting process, a signal $x\left(t\right)$ or function $f\left(t\right)$ can be expanded or decomposed as follows:

(5) $$f\left(t\right)=\sum_{i=1}^{n}c_{i}+r_{n},$$

where $c_{i}$ represents the intrinsic mode functions and $r_{n}$ is a residue.

Overall, $c_{1}$ should contain the highest-frequency component of the signal; $c_{n}$ should contain the lowest-frequency component; and $r_{n}$ should be either a trend or a constant.

In the case of DE, a *d*-dimensional optimization problem was considered. In our experiments, we used a population size, *n*, of solution vectors, $x_{i}$, i=1, 2,…, n, to search for the optimal solution. These solutions can be represented by $x_{j,i}$, j=1, 2,…, d (the dimension of a solution is d) and i=1, 2,…, n. For generation *t*, the solution vector $x_{i}$ is denoted by $x_{i}^{t}$. Initially, $x_{i}^{0}$ values are uniformly generated between the lower bound of the domain, $x_{\min}$ and the upper bound, $x_{\max}$.

Typically, a DE algorithm [24,25,26,27,28,29] contains three main steps: mutation, crossover and selection. In the step of mutation, three random independent indices $r_{1}$, $r_{2}$ and $r_{3}$ ranging from 1 to n are first selected—i.e., $r_{1}\neq r_{2}\neq r_{3}$—and their corresponding solution vectors, $x_{r_{1}}$, $x_{r_{2}}$ and $x_{r_{3}}$ are then obtained. Finally, a mutant vector [26] or a donor vector [27] is generated,

(6) $$v_{i}^{t+1}=x_{r_{1}}^{t}+F\left(x_{r_{2}}^{t}-x_{r_{3}}^{t}\right),$$

where *F*, the differential weight, is a positive real number that normally lies between 0 and 1. In addition, these three random independent indices also meet the constraint $r_{1}\neq r_{2}\neq r_{3}\neq i$.

In the crossover step, a crossover vector or a trial vector [26] via the binominal scheme is obtained as follows:

(7) $$u_{j,i}^{t+1}=\left\{\begin{array}{l} v_{j,i}^{t+1}\;\mathrm{if}\;r_{j,i}\leq C_{r}\;\mathrm{or}\;j=I_{j}^{r}\\ x_{j,i}^{t}\;\mathrm{otherwise} \end{array}\right.,$$

where $C_{r}$ is the crossover rate and $I_{j}^{r}$ is a random index, i.e., an integer ranging from 1 to *d*.

In the step of selection, a greedy scheme is adopted. For our minimization problem, this is mathematically described as follows:

(8) $$x_{i}^{t+1}=\left\{ \begin{array}{l} u_{i}^{t+1}\;\mathrm{if}\;f\left(u_{i}^{t+1}\right)\leq f\left(x_{i}^{t}\right)\\ x_{i}^{t}\;\mathrm{otherwise} \end{array}\right..$$

For the purpose of this study, the fitness or objective function $f\left(x\right)$ is the MSE between the source signal and the searched signal, i.e., a combination of searched waves.

## 3. Problem Formulation

Even EEMD can deal with some mode-mixing problems of EMD, but it cannot decompose some source signals with a combination of single or complex waveforms into separate components. The main reason for this is that EMD and EEMD act essentially as a dyadic filter bank [21,22,23]. Therefore, when these single or complex waveforms lie within the same dyadic filter bank, they cannot be well decomposed into separate components; even for the source signals with a combination of simple periodic waves, EMD-like data-driven methods cannot decompose them well, which is especially true for non-continuous waves.

Therefore, the main purpose of this paper is to propose an effective technique to decompose this kind of source signal into separate components. A feasible approach is to directly rather than indirectly use nature-inspired optimization algorithms. In this paper, DE is adopted because it is a real-coded algorithm, which is much more efficient than a binary-coded algorithm; binary-coded algorithms need additional computational time to transform binary code into real code and vice versa. An appropriate objective function plays a very important role in optimization algorithms. In this work, we choose the MSE between a source signal and its corresponding searched signal as our objective function. Therefore, the source signals and their corresponding objective functions are presented in the following subsections.

### 3.1. Source Signals

As a pioneering work, this paper only considers four simple yet representative source signals, consisting of three combinations of single waves and one combination of composite waves. These three single waves are sinusoidal, square, and triangular waves; the remaining one is a composite of the previous three single waves. Each source signal is composed of three components. Based on each individual source wave, the objective function is defined accordingly.

#### 3.1.1. Sinusoidal Waves

As components of the Fourier transform [2], sinusoidal waves are the most representative of signals. Thus, the first source signal is a signal synthesis of three sinusoidal waves as follows:

(9) $$s\left(t\right)=\sum_{k=1}^{3}a_{k}\sin\left(2\pi f_{k}t+p_{k}\right),$$

where the $a_{k}$ values for k=1, 2, 3, are amplitudes; $f_{k}$ values are normalized frequencies (simply called frequencies in the following) lying between 0 and 1; $p_{k}$ values are phases lying between 0 and 2π; and t is an integer from 0 to L−1, where L is the length of the signal series.

#### 3.1.2. Square Waves

The second signal source is a signal synthesis of three square waves as follows:

(10) $$s\left(t\right)=\sum_{k=1}^{3}a_{k}\mathrm{square}\left(2\pi f_{k}t+p_{k}\right),$$

where the definitions of the $a_{k}$, $f_{k}$, and $p_{k}$ values for k=1, 2, 3 as well as t are the same as those of Equation (9).

#### 3.1.3. Triangular Waves

The third signal source is a signal synthesis of three triangular waves as follows:

(11) $$s\left(t\right)=\sum_{k=1}^{3}a_{k}\mathrm{triangle}\left(2\pi f_{k}t+p_{k}\right).$$

Likewise, the definitions of all symbols are the same as those of Equation (9).

#### 3.1.4. Composite Waves

The fourth signal source is a signal synthesis of the previous three waves as follows:

(12) $$s\left(t\right)=a_{1}\sin\left(2\pi f_{1}t+p_{1}\right)+a_{2}\mathrm{square}\left(2\pi f_{2}t+p_{2}\right)+a_{3}\mathrm{triangle}\left(2\pi f_{3}t+p_{3}\right).$$

The definitions of all symbols are also the same as those of Equation (9).

### 3.2. Objective Function

The formulas of Equations (9)–(12) indicate that every wave consists of three parameters—the amplitude, frequency and phase—and thus each source signal contains nine unknown parameters; or its dimension is nine, i.e., d=9. Therefore, we set the $x_{i}$, i=1, 2,…, 9 as our searched parameters, and $z\left(t\right)$ as the searched signal:

(13) $$z\left(t\right)=x_{1}\sin\left(2\pi x_{2}t+x_{3}\right)+x_{4}\sin\left(2\pi x_{5}t+x_{6}\right)+x_{7}\sin\left(2\pi x_{8}t+x_{9}\right).$$

It is reasonable and effective for the objective function to be defined as the MSE between the source signal and the searched signal as follows:

(14) $$f\left(x\right)=\frac{1}{L}\sum_{t=0}^{L-1}{\left(s\left(t\right)-z\left(t\right)\right)}^{2},$$

where $s\left(t\right)$ is taken from Equation (9).

For other source signals, their objective functions are defined accordingly.

## 4. Experimental Results and Discussion

In this section, experimental parameters for DE are set, including the differential weight, the crossover rate, the dimension and the population size. Afterwards, our experiments are presented and then the corresponding experimental results are discussed.

### 4.1. Experimental Settings

Since each source signal consisted of nine parameters, its dimension of DE was nine, i.e., d=9. The amplitude of the first component was set to be 1, the frequency 0.01 and the phase 0; the amplitude of the second component was to be 2, the frequency 0.02 and the phase 0.125π; the amplitude of the third component was to be 3, the frequency 0.03 and the phase 0.25π. For clarity, the first source signal is represented as follows:

(15) $$s\left(t\right)=\sin\left(2\pi \left(0.01\right)t+0\right)+2\sin\left(2\pi \left(0.02\right)t+0.125\pi \right)+3\sin\left(2\pi \left(0.03\right)t+0.25\pi \right).$$

The population size, n, of DE was set to be 90, i.e., n=90; each experiment was run 20 times, and the maximum number of iterations—the termination criterion—was set to be 15,000 for each run. In addition, the differential weight was set to be 0.5, and the crossover rate was 0.1.

For the domains of the amplitude, frequency and phase parameters, it is much easier to set; a good choice for the amplitude is the difference between the maximum and minimum values of the source signal, which is sufficient to cover all potential amplitudes. With the help of DFT, a better choice for the amplitude is two times the maximum value of all frequency amplitudes, which is sufficient to cover all potential amplitudes in a smaller range.

For normalized frequencies, the domain of frequency, [0, 0.5], and the domain of phase, [0, 2π], together will cover all possibilities of frequency and phase. Therefore, the domains of the amplitude, frequency, and phase were set to be [0, 10], [0, 0.5] and [0, 2π], respectively. All initial positions of each population were randomly and uniformly distributed in the search space.

During the search process, all members of the population were restricted in the search space; when any member of the population flew out of the domain, it was replaced by the upper or lower limit of the domain according to its solution. The operation was implemented by a clamping function of the position, lying between $x_{\min}$ and $x_{\max}$, where $x_{\min}$ is the lower limit and $x_{\max}$ the upper limit.

### 4.2. Experimental Results

In the following subsections, four source signals are considered, consisting of three combinations of single waves and one combination of three waves. These three single waves are sinusoidal, square and triangular waves; the composite wave is composed of the previous three single waves.

#### 4.2.1. Sinusoidal Waves

**Figure 1:**
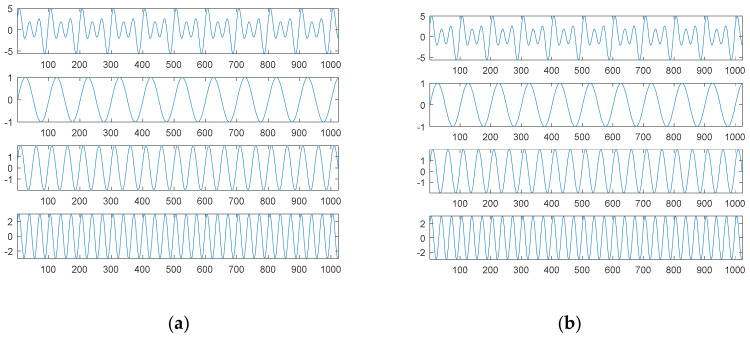
The first source signal: (**a**) the source signal and its three individual components; (**b**) the searched components and their combination (top).

The first source signal, as in Equation (15), consisted of a combination of three sinusoidal waves, which were common continuous and smooth functions. Figure 1 shows the results through SDDE.

The first graph (in the first panel) of Figure 1a is the first source signal; the second graph (in the second panel) is the first original sinusoidal wave, $\left(a_{1},\;f_{1},\;p_{1}\right)=\left(1,\;0.01,\;0\right)$; the third graph (in the third panel) is the second original sinusoidal wave, $\left(a_{2},\;f_{2},\;p_{2}\right)=\left(2,\;0.02,\;0.125\pi \right)$; and the fourth graph (in the fourth panel) is the third original sinusoidal wave, $\left(a_{3},\;f_{3},\;p_{3}\right)=\left(3,\;0.03,\;0.25\pi \right)$.

In order to understand the feasibility and potential of SDDE, we only show a plot with the best performance, i.e., the minimum error among 20 runs. Even in the primary stage, Table 1 shows that two runs can still achieve the true minimum error; therefore, the searched parameters of Run 7 were fetched to generate Figure 1b.

Obviously, Figure 1b shows the same results as Figure 1a; that is, we can find three exact components through SDDE. It is worth noting that even if we can determine the exact components, the order of appearance from the source signal is usually different from that from searched signal.

Except for two runs with the best zero error, Table 1 also shows the MSEs of 12 runs that were close to 0, while the other six runs had bad performance.

**Figure 2:**
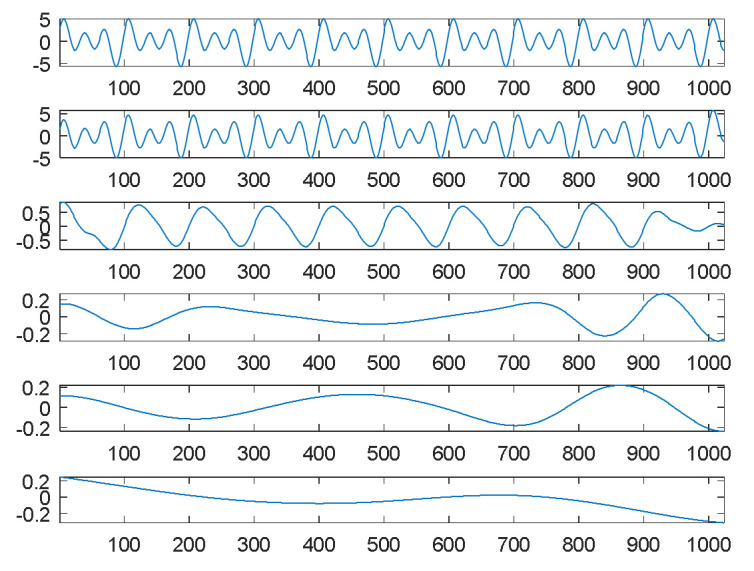
Empirical mode decomposition of the first source signal: the source signal, IMF1–IMF4 and residue from top to bottom.

On the surface, it seems to be easy to decompose this smooth signal into separate components. For comparison, EMD was adopted to perform the decomposition task. Under the same source signal, its decomposition results are shown in Figure 2. The first graph of Figure 2 is the source signal; the second is the first IMF, simply denoted by IMF1; the third to fifth graphs are IMF2 to IMF4; and the final graph is the residue.

Compared to our proposed SDDE approach, Figure 2 shows that the waveforms of graphs 1 and 2 (the source signal and the highest-frequency component) are almost the same except for their amplitudes and boundaries. From another perspective, EMD automatically views the source signal as a special high-frequency component (IMF1), and its special low-frequency component (IMF2) is similar to a sinusoidal function. The results also reflect the fact that EMD acts essentially as a dyadic filter bank [21,22,23]. Therefore, some compound waves, even sinusoidal waves, cannot further be decomposed into separate waves when these components lie in the same dyadic filter bank.

In this case, the special composite high-frequency component consists of three sinusoidal waves. Because of the inherent characteristics of EMD, it cannot further decompose the special composite high-frequency component into separate components.

#### 4.2.2. Square Waves

The second source signal consisted of a combination of three square waves, which were non-continuous and also non-smooth functions. Their amplitudes, frequencies and phases were the same as those of the sinusoidal waves. The formula of the second source signal is almost the same as Equation (15), except that the sinusoidal function is replaced by its corresponding square function.

**Figure 3:**
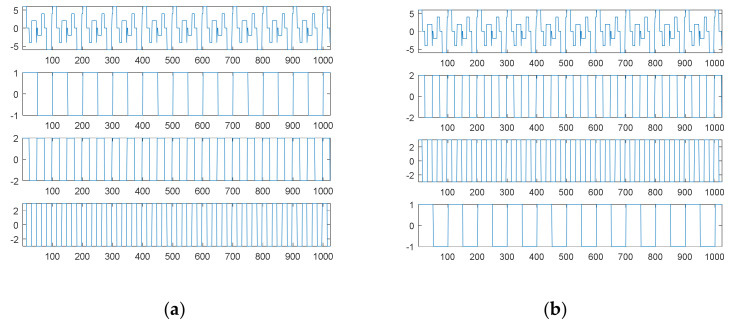
The second source signal: (**a**) the source signal and its three individual components; (**b**) the searched components and their combination (top).

Figure 3a shows the second source signal and its separate waves; Figure 3b shows the searched components through SDDE and their combination. Likewise, these graphs were produced based on the first set of parameters with the best performance, i.e., the parameters of Run 1.

Generally, non-continuous functions are difficult to decompose; however, this task is easy for our proposed SDDE. In fact, Table 1 shows that all 20 runs can achieve the true minimum error. Therefore, we can determine three exact components through SDDE.

Figure 3b shows almost the same waves as Figure 3a except for the order of appearance. The change of the order of appearance is due to the randomness of DE.

**Figure 4:**
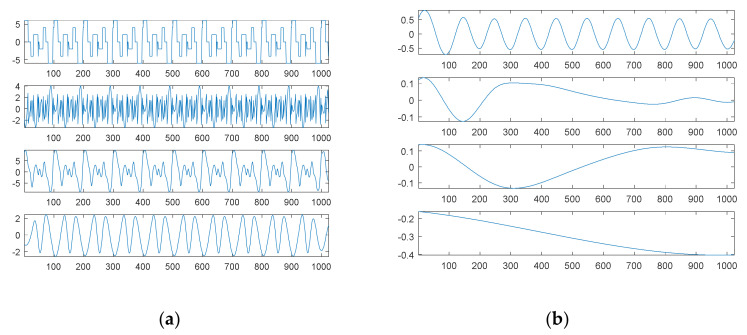
Empirical mode decomposition of the second source signal: (**a**) the source signal, IMF1–IMF3 from top to bottom; (**b**) IMF4–IMF6 and residue from top to bottom.

For comparison, EMD was used to decompose the second source signal; the decomposition results, consisting of eight panels or graphs, are shown in Figure 4. For visual consideration, Figure 4 is composed of two plots (Figure 4a,b); the four graphs of Figure 4a are the source signal and IMF1 to IMF3, from top to bottom; the four graphs of Figure 4b are IMF4 to IMF6 and the residue from top to bottom.

As expected, EMD cannot decompose the source signal into three square waves well. Since the square waves are non-continuous and also non-smooth functions, EMD produces an extremely complicated highest-frequency component (IMF1) in response to non-continuity and also non-smoothness. Except for the potential end effects of EMD, IMF2 to IMF3 can be viewed as two special periodic components; IMF4 is a relatively low-frequency component similar to a sinusoidal function.

Although EMD can decompose some hidden signal patterns in the case of a combination of periodic waves, these signal patterns are generally compound waves within the same dyadic filter bank [21,22,23], and they are therefore difficult to recognize and explain. For better understanding, it is necessary to further decompose them into separate signal patterns. Obviously, our proposed SDDE approach is much better than EMD in this case.

#### 4.2.3. Triangular Waves

The third source signal consisted of a combination of three triangular waves, which were continuous but non-smooth functions. Likewise, their amplitudes, frequencies and phases were the same as those of the sinusoidal waves. The formula of the third source signal is almost the same as Equation (15) except that the sinusoidal function is replaced by its corresponding triangular function.

**Figure 5:**
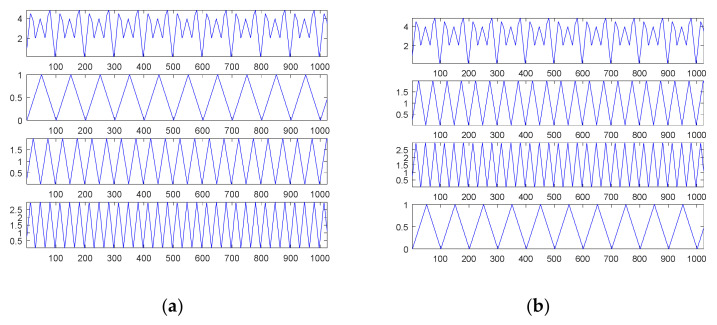
The third source signal: (**a**) the source signal and its three individual components; (**b**) the searched components and their combination (top).

Figure 5a shows the third source signal and its separate waves; Figure 5b shows the searched components through SDDE and their combination. These graphs were also produced based on the first set of parameters with the best performance, i.e., the parameters of Run 9.

Table 1 shows that Run 9 exhibited the best performance. Except for tolerable computational error, we were almost able to determine three exact components through SDDE, and hence its result was chosen to generate Figure 5b. Not surprisingly, Figure 5b shows almost the same waves as Figure 5a except for the order of appearance.

Compared to the previous two source signals, Table 1 also shows that the third source signal is more difficult to search or decompose into its exact signal components. Even though only one of 20 runs exhibited close to zero error, SDDE shows potential for perfectly decomposing source signals.

**Figure 6:**
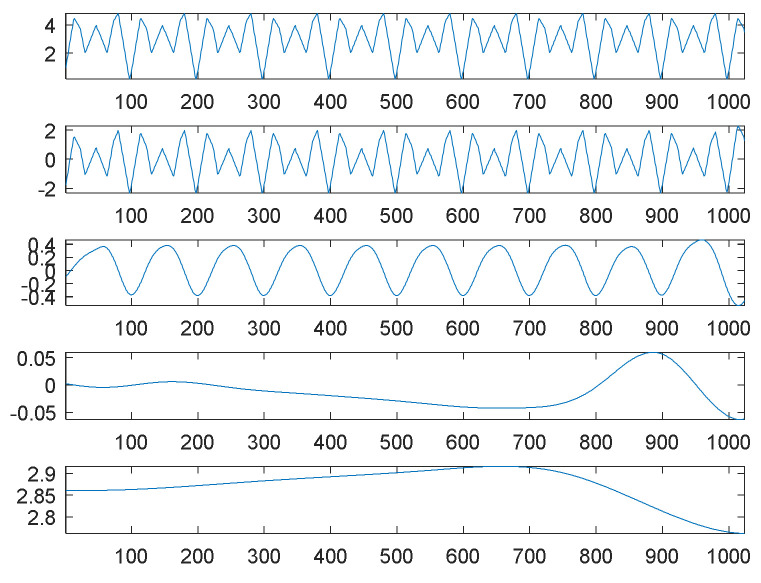
Empirical mode decomposition of the third source signal: the source signal, IMF1–IMF4 and residue from top to bottom.

Figure 6 shows the decomposition results of the third source signal by EMD. Similar to the first source signal, EMD essentially views the source signal as an intrinsic mode function, and thus it can only decompose the source signal into two main components: one is a scaled version of the source signal (IMF1), and the other is almost a sinusoidal function (IMF2). The results also reflect the fact that EMD acts essentially as a dyadic filter bank [21,22,23]. Therefore, some compound waves, even triangular waves, cannot further be decomposed into separate waves when these components lie in the same dyadic filter bank.

#### 4.2.4. Composite Waves

The fourth source signal consisted of a combination of three different waves, which were also non-continuous and non-smooth functions. The first wave was the sinusoidal wave; the second was the square wave; and the third was the triangular wave. The amplitude, phase and frequency were the same as those of the first source signal. For clarity, the fourth source signal is represented as follows:

(16) $$s\left(t\right)=\sin\left(2\pi \left(0.01\right)t+0\right)+2\mathrm{square}\left(2\pi \left(0.02\right)t+0.125\pi \right)+3\mathrm{triangle}\left(2\pi \left(0.03\right)t+0.25\pi \right).$$

**Figure 7:**
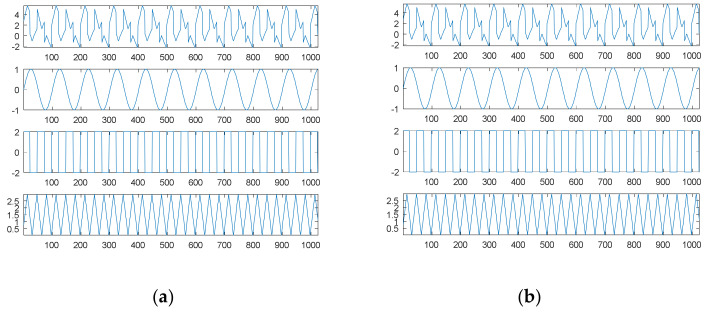
The fourth source signal: (**a**) the source signal and its three individual components; (**b**) the searched components and their combination (top).

Figure 7a shows the fourth source signal and its separate waves; Figure 7b shows the searched components through SDDE and their combination. Likewise, these graphs were produced based on the first set of parameters with the best performance, i.e., the parameters of Run 3.

Table 1 shows that Run 3 could achieve the true minimum error; thus, SDDE was able to decompose the source signal into three exact components. Obviously, Figure 7b shows the same waves as Figure 7a.

**Figure 8:**
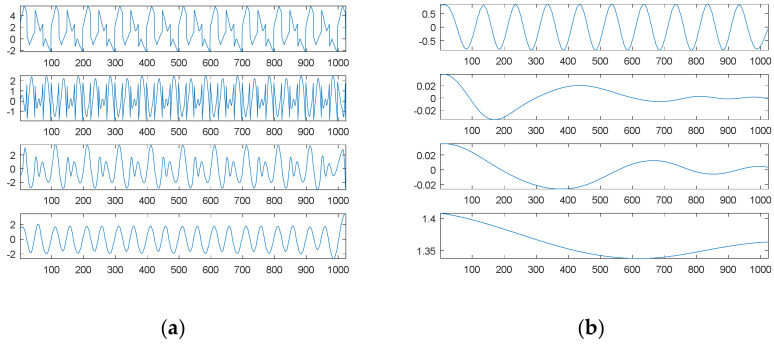
Empirical mode decomposition of the fourth source signal: (**a**) the source signal, IMF1–IMF3 from top to bottom; (**b**) IMF4–IMF6 and residue from top to bottom.

Similar to the second source signal, EMD cannot decompose the source signal into three separate waves. Its decomposition results are shown in Figure 8. For visual consideration, Figure 8 is composed of two plots (Figure 8a,b): the four graphs of Figure 8a are the source signal and IMF1 to IMF3 from top to bottom, and the four graphs of Figure 8b are IMF4 to IMF6 and the residue from top to bottom.

As with the second source signal, the fourth source signal contains a non-continuous square wave and a non-smooth triangular wave. Therefore, EMD also produces an extremely complicated highest-frequency component (IMF1) in response to non-continuity and non-smoothness. Likewise, IMF2 is also viewed as one special periodic component; except for the potential end effects, IMF3 and IMF4 are two relatively low-frequency components similar to a sinusoidal function; the frequency of IMF3 is about twice that of IMF4.

In the case of the fourth source signal, our proposed SDDE approach can directly decompose the source signal into three different waves, but EMD only can provide some hidden characteristics which need further analysis and explanations.

#### 4.2.5. Signal Decomposition in the Presence of Noise

To show that our proposed SDDE approach can work in practical applications, in the following two experiments, we added 10 dB white noise to each source signal; the signal-to-noise ratio (SNR) [30] is defined by:

(17) $$SNR=10\log_{10}\left(P_{s}/P_{n}\right),$$

where

(18) $$P_{s}=\frac{1}{N}\sum_{t=0}^{N-1}s^{2}(t)$$

and the variance of the white noise is as follows:

(19) $$P_{n}=\sigma_{n}^{2}.$$

After we compute the power, $P_{s}$, of the signal, the standard deviation of the white noise can be computed by the following formula:

(20) $$\sigma_{n}=\sqrt{P_{s}/10^{SNR/10}}.$$

In the first experiment, we only knew the exact type of hidden signal components but did not know how many components existed. Since the dimension of true signal components was nine (three signal components, each with three parameters), we supposed that there were six signal components, and hence the dimension was a total of 18. Likewise, the population size, n, was set to be 90.

Table 2 lists four experimental results, where $P_{r}$ is the power of real noise. In the presence of noise, zero error is impossible. The error for each run will reflect the performance of SDDE; the closer the value to $P_{r}$, the better the performance. These results show that SDDE could still determine hidden signal components in the presence of noise in all four cases. 

**Figure 9:**
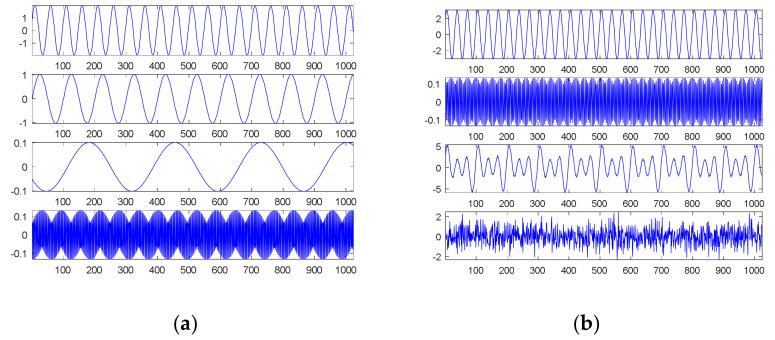
The searched components of the first noised source signal (10 dB white noise): (**a**) four possible sinusoidal signal components; (**b**) two possible sinusoidal signal components, a combination of six possible sinusoidal components and the residue from top to bottom.

To visualize the data in a limited space, we only show the decomposed results of the first source signal—a combination of three sinusoidal waves—in Figure 9. The first six graphs of Figure 9 show six possible sinusoidal waves, respectively. The first graph of Figure 9a is equivalent to the second original sinusoidal wave; the second graph is equivalent to the first original sinusoidal wave. The first graph of Figure 9b is equivalent to the third original sinusoidal wave. The magnitudes of the other three possible sinusoidal waves were all lower than 0.15, which were in fact artifacts due to white noise. The third graph of Figure 9b is a combination of six possible sinusoidal components, which is quite similar to the first source signal; the residue is in the fourth graph.

**Figure 10:**
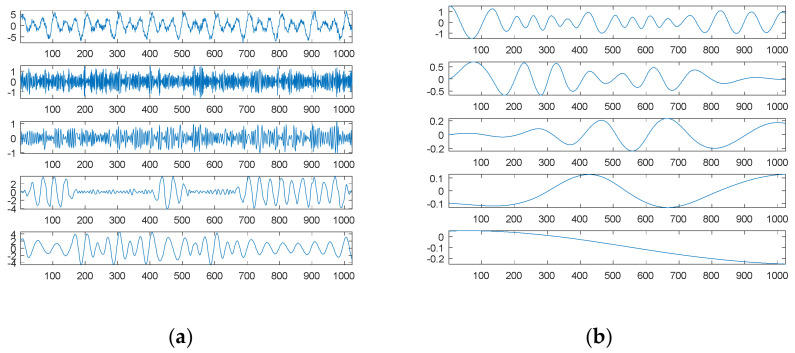
Empirical mode decomposition of the first noised source signal (10 dB white noise): (**a**) the source signal with noise, IMF1–IMF4 from top to bottom; (**b**) IMF5–IMF8 and residue from top to bottom.

For comparison, Figure 10 shows the decomposition results through EMD of the first noised source signal (10 dB white noise); Figure 10a contains the first noised source signal and IMF1 to IMF4 from top to bottom; and Figure 10b contains IMF5 to IMF8 and the residue from top to bottom. Unlike our proposed SDDE approach, EMD is easily affected by white noise; no separate sinusoidal wave could be decomposed by EMD under the contaminated source signal.

In the second experiment, we only knew that hidden signal components were composed of three types of sinusoidal, square and triangular waves but did not know how many components existed. Since the dimension of true signal components was nine, we supposed that there were nine signal components and hence the dimension was a total of 27. In addition, the population size, n, was set to be 135.

Table 3 lists three experimental results. Since the error for each run was closer to the power of real noise, $P_{r}$, the table shows that SDDE could determine hidden signal components even in the presence of noise in all three cases. 

**Figure 11:**
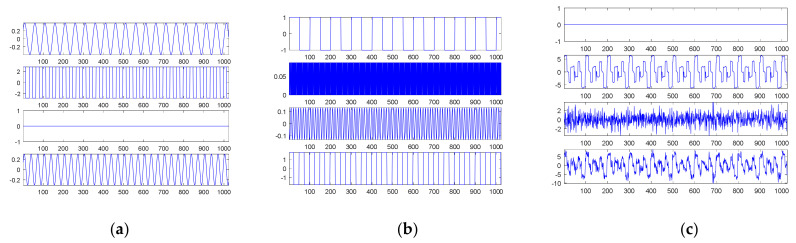
The searched components of the second noised source signal (10 dB white noise): (**a**) possible sinusoidal, square, triangular and sinusoidal signal components from top to bottom; (**b**) possible square, triangular, sinusoidal and square signal components from top to bottom; (**c**) a possible triangular signal component, a combination of nine possible components, a residue and the second noised source signal from top to bottom.

Likewise, due to a limited space, we only visualize the decomposed results of the second source signal—a combination of three square waves—in Figure 11. The first nine graphs of Figure 11 in turn show the sinusoidal, square and triangular waves, respectively. The second graph of Figure 11a is equivalent to the third original square wave; the third graph is 0; and the amplitudes of the other two graphs are lower than 0.4, which are in fact artifacts due to white noise.

The first graph of Figure 11b is equivalent to the first original square wave; the fourth graph shows the second original square wave; and the amplitudes of the other two graphs are lower than 0.15, which are in fact artifacts due to white noise. The amplitude of the first graph of Figure 11c is zero; the second graph shows a combination of nine possible square components, which is quite similar to the second source signal; a residue is shown in the third graph; and the fourth graph shows the second noised source signal.

**Figure 12:**
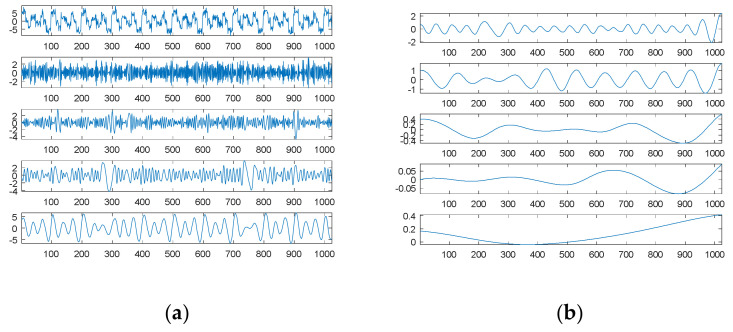
Empirical mode decomposition of the second source signal with 10 dB white noise: (**a**) the source signal with noise, IMF1–IMF4 from top to bottom; (**b**) IMF5–IMF8 and residue from top to bottom.

For comparison, Figure 12 shows the decomposition results through EMD of the second noised source signal; Figure 12a contains the second noised source signal and IMF1 to IMF4 from top to bottom; Figure 12b contains IMF5 to IMF8 and the residue from top to bottom. Unlike our proposed SDDE approach, EMD is easily affected by white noise; no separate square wave can be decomposed by EMD under the contaminated source signal.

#### 4.2.6. Signal Decomposition through Discrete Fourier Transform

**Figure 13:**
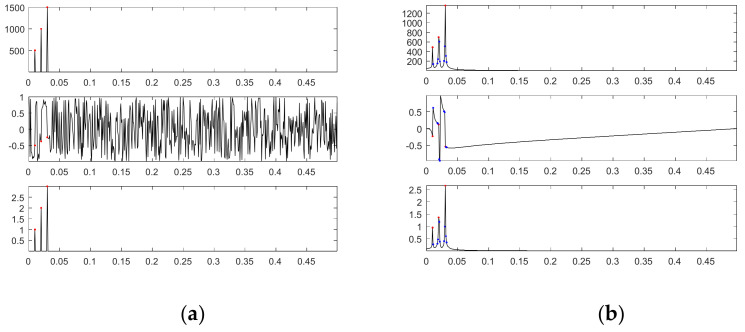
Amplitude spectrum and phase spectrum of the first source signal through DFT as well as the corresponding sinusoidal amplitude plot (from top to bottom): (**a**) *L* = 1000; (**b**) *L* = 1024.

To understand the differences between our proposed SDDE approach and DFT, Figure 13a shows the amplitude spectrum and phase spectrum through DFT with *L* = 1000 of the first source signal as well as the plot of the corresponding sinusoidal amplitudes; Figure 13b shows the corresponding plots for *L* = 1024. In the phase spectrums, the magnitudes are normalized by π for easy comparison. Since DFT has a frequency resolution of 1/*L*, *L* = 1000 can make our adopted three frequencies (0.01, 0.02 and 0.03) match the resolution, but *L* = 1024 cannot. In Figure 13, the points marked in red represent three main components closer to the original three frequencies; except for the previous three main components, the points marked in blue represent other main components that their amplitudes are 0.1 times larger than the maximum value of all amplitudes.

**Figure 14:**
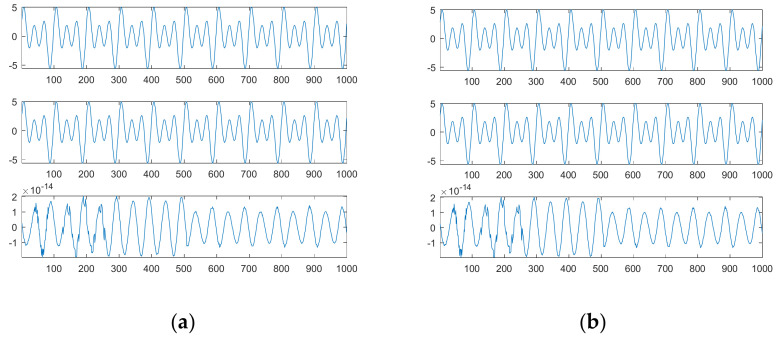
The source signal, the reconstructed signal and the corresponding signal error (from top to bottom) for *L* = 1000: (**a**) three main components; (**b**) all main components.

For *L* = 1000, Figure 13a obviously shows that there are only three main frequency components; they are exactly 0.01, 0.02 and 0.03; their corresponding phases are −0.5π, −0.375π and −0.25π; their corresponding sinusoidal amplitudes are 1, 2 and 3. Since the fundamental basis function of DFT for reconstruction is the Cosine function, their corresponding phases for the Sine function need to be right shifted by 0.5π; the shifted phases are 0, 0.125π and 0.25π, which are the same as the phases of the first source signal. Figure 14a shows from top to bottom the first source signal, the reconstructed signal with three main components and the corresponding signal error; Figure 14b shows the corresponding plots for the reconstructed signal with all main components.

Since our adopted three frequencies match the resolution, DFT can accurately decompose the first source signal into three main components; therefore, there is no difference between the reconstructed signal with three main components and that with all main components.

For *L* = 1024, Figure 13b shows that there are more than three main frequency components. We chose as our three main components three frequencies of 0.0098, 0.0195 and 0.0303, which are closer to three frequencies of the first source signal; their corresponding phases are −0.2282π, 0.1193π and −0.5349π for the Cosine function or 0.2718π, 0.6193π and −0.0349π for the Sine function, which are much different from the corresponding original phases (0, 0.125π and 0.25π); their corresponding sinusoidal amplitudes (0.9493, 1.3639 and 2.6559) are also a little different from the corresponding original amplitudes (1, 2 and 3).

**Figure 15:**
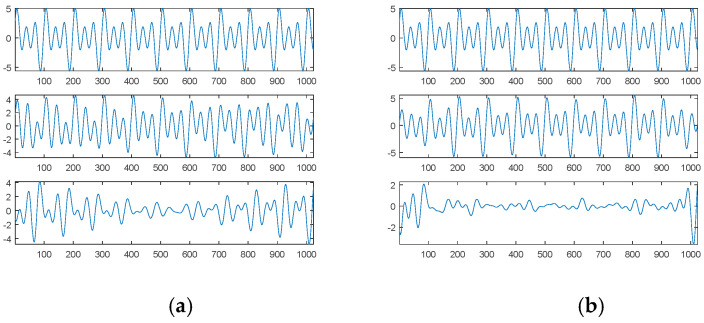
The source signal, the reconstructed signal and the corresponding signal error (from top to bottom) for *L* = 1024: (**a**) three main components; (**b**) all main components.

Figure 15a shows the first source signal, the reconstructed signal with three main components and the corresponding signal error; Figure 15b shows the corresponding plots for the reconstructed signal with all main components.

Due to a finite frequency resolution, DFT cannot accurately determine three components. Therefore, the error between the first source signal and the reconstructed signal with three main components is much significant; even all main components are considered, the error is also significant. In contrast, our proposed SDDE approach can exactly determine the three main components.

**Figure 16:**
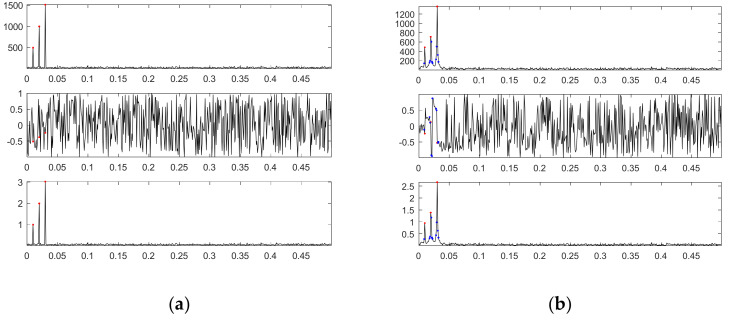
Amplitude spectrum and phase spectrum of the first source signal contaminated by 10 dB white noise through DFT as well as the corresponding sinusoidal amplitude plot (from top to bottom): (**a**) *L* = 1000; (**b**) *L* = 1024.

In the presence of 10 dB white noise, Figure 16a shows the amplitude spectrum and phase spectrum of the first noised source signal through DFT with *L* = 1000 as well as the corresponding sinusoidal amplitudes plot; Figure 16b shows the corresponding plots for *L* = 1024.

For *L* = 1000 and in the presence of 10 dB white noise, Figure 16a shows that there are also only three main frequency components; they are exactly 0.01, 0.02 and 0.03; their corresponding phases are −0.5081π, −0.3734π and −0.2536π for the Cosine function, or −0.0081π, 0.1266π and 0.2464π for the Sine function, which are close to the phases of the first source signal; their corresponding sinusoidal amplitudes are 0.9884, 2.0958 and 3.0135, which are close to the amplitudes of the first source signal.

**Figure 17:**
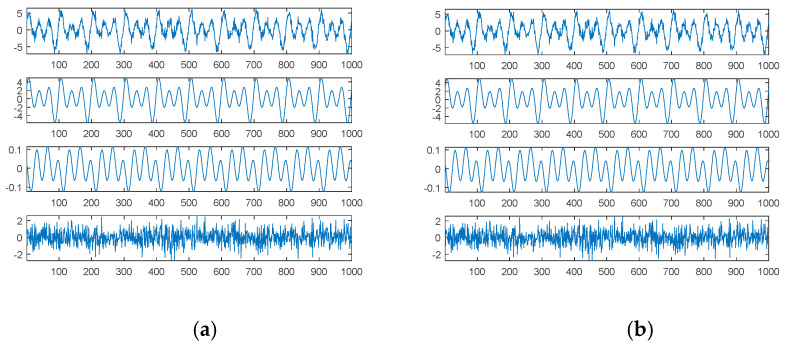
The first noised source signal (10 dB white noise), the reconstructed signal, the error between the reconstructed signal and the first source signal, and the error between the reconstructed signal and the first noised source signal (from top to bottom) for *L* = 1000: (**a**) three main components; (**b**) all main components.

Figure 17a shows from top to bottom the first noised source signal, the reconstructed signal with three main components, the error between the reconstructed signal and the first source signal, and the error between the reconstructed signal and the first noised source signal. Figure 17b shows the corresponding plots for the reconstructed signal with all main components.

For *L* = 1024 and in the presence of 10 dB white noise, Figure 16b shows that there are more than three main frequency components. We chose as our three main components three frequencies of 0.0098, 0.0195 and 0.0303, which are closer to three frequencies of the first source signal; their corresponding phases are −0.2168π, 0.1303π and −0.5331π for the Cosine function or 0.2832π, 0.6303π and -0.0331π for the Sine function, which are much different from the corresponding original phases (0, 0.125π and 0.25π); their corresponding sinusoidal amplitudes (0.9500, 1.3752 and 2.6818) are also much different from the corresponding original amplitudes (1, 2 and 3).

**Figure 18:**
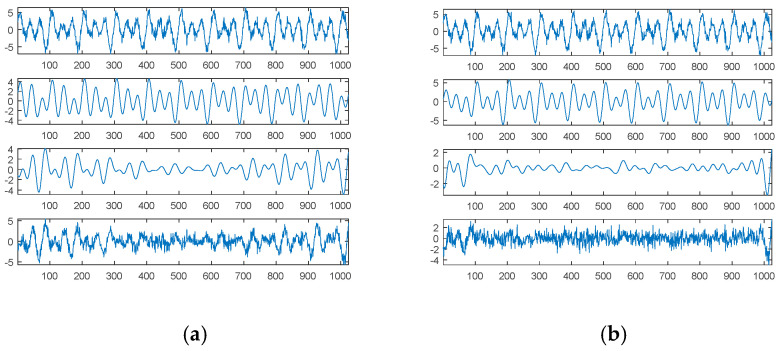
The first noised source signal (10 dB white noise), the reconstructed signal, the error between the reconstructed signal and the first source signal, and the error between the reconstructed signal and the first noised source signal (from top to bottom) for *L* = 1024: (**a**) three main components; (**b**) all main components.

Figure 18a shows from top to bottom the first noised source signal, the reconstructed signal with three main components, the error between the reconstructed signal and the first source signal, and the error between the reconstructed signal and the first noised source signal. Figure 18b shows the corresponding plots for the reconstructed signal with all main components.

Likewise, DFT cannot accurately determine three components due to a finite frequency resolution. Therefore, the error between the first source signal and the reconstructed signal with three main components is much significant; even all main components are considered, the error is also significant. In contrast, our proposed SDDE approach can determine the three main components at a tolerable level.

### 4.3. Discussion

Through EMD, the currently most widely-used data-driven decomposition tool, we can determine some regular patterns for signals with hidden periodic functions. The number of all possible signal components, IMFs, depends on the complexity of the source signal; it is normally a fraction of the length of the source signal. Hence, EMD has a potential problem of frequency resolution.

For smooth and non-smooth continuous signals with hidden periodic functions, EMD generally produces sinusoidal patterns with deformations in the boundaries because of end effects; in addition, it also produces some special periodic signal patterns with little deformation in the boundaries, which can be viewed as its unique basis functions, while some are a combination of several standard basis functions, such as sinusoids.

For non-continuous signals with hidden periodic functions, it also produces some special periodic signal patterns with little deformation in the boundaries, which fluctuate wildly depending on how many non-continuous components the source signal contains. For example, a combination of three square waves varies more wildly than a composite of three different waves because the former contains more non-continuous components than the latter.

Because of the inherent characteristics of EMD—i.e., the fact that EMD acts essentially as a dyadic filter bank [21,22,23]—it cannot further decompose some compound waves, even sinusoidal waves, into separate waves when these compound waves lie in the same dyadic filter bank. In contrast, our proposed SDDE approach can perfectly or almost perfectly decompose any compound waves into separate waves when the types of waves and the number of these types are known. Even in the presence of 10 dB white noise, our proposed SDDE approach still determine the main signal components when one or three types of waves are known but without knowing the number of these types.

On the other hand, based on the basis functions of the Sine function or Cosine function, DFT can also decompose any signal into a combination of the basis functions. The number of all possible signal components shown by its amplitude spectrum is the length of the source signal. Hence, DFT also has a potential problem of frequency resolution, but less severer than that of EMD.

For the first source signal, a combination of sinusoidal waves, if the frequencies of the original signal components match the resolution, then DFT can accurately determine three main components even in the presence of 10 dB white noise; but if the frequencies of the original signal components do not match the resolution, DFT decomposes the signal into more than three main components, causing a big error between the reconstructed signal and the source signal. As for the other three source signals or combinations of other periodic waves, DFT cannot decompose well even for signals without noise contamination. In contrast, our proposed SDDE approach perform well ever for signals with 10 dB white noise.

EMD and DFT both can quickly decompose any signal into possible signal components; through IMFs, EMD shows possible composites of single components of a signal; through amplitude spectrum, DFT shows possible single components of a signal. Even in the presence of 10 dB white noise and with a finite frequency resolution, EMD always shows possible patterns of a signal; DFT always shows possible components of a signal. The importance of an IMF is proportional to its magnitude; likewise, the importance of the sinusoidal wave at a certain frequency is proportional to the magnitude of the frequency spectrum at the frequency. The components with relative large magnitudes are called the main components.

Due to a considerably finite frequency resolution, EMD easily causes frequency mixing, i.e., an IMF consisting of different frequency components. Although DFT has a larger frequency resolution than EMD, its resolution is also finite. In contrast, our proposed SDDE approach has an infinite frequency resolution, and hence it has the opportunity to exactly decompose.

In addition, EMD and DFT both cannot do well in the signals of non-smooth waves; for EMD, how to explain the decomposed components is a big challenge; for DFT, the basis functions are always sinusoidal waves. In contrast, our proposed SDDE approach can adopt various periodic waves as our basis functions.

Contrary to EMD and DFT, it is necessary for our proposed SDDE approach to first decide what types of signal components are searched, and therefore how to determine the types of hidden signal components is a big challenge. Except for domain knowledge and individual experience, the main components of EMD and DFT can provide much information about types of signal components and their corresponding numbers.

In the current stage, our proposed SDDE approach focuses on three common periodic signals, but it can be easily applied to complex signals, such as chirp signals [31], or aperiodic signals, such as aperiodic rectangular pulses, or radar signals [32], or amplitude- and frequency-modulated (AM and FM) component signals [6], as long as their signal features can be described by formulas. When source signals are complicated and even contaminated by noise, it is usually necessary to adopt an advanced optimization algorithm combined with domain knowledge and the information provided by DFT and EMD.

DFT provides all possible single components, and therefore, DFT performs well in the signals with sinusoidal waves; EMD provides possible simple and complex combinations of all single components, and therefore EMD performs well in the signals with AM and FM waves. As EMD provides an auxiliary tool to help DFT obtain and explain some complex signal waveforms, our proposed SDDE approach provides an auxiliary tool to help DFT obtain more accurate information about signals with combinations of sinusoidal signals, and can also decompose signals with combinations of various periodic waves.

## 5. Conclusions

In this paper, a novel signal decomposition technique, called signal decomposition by differential evolution (SDDE), was proposed to decompose various periodic signals. At the pioneering stage, we only considered four common periodic signals, which were a combination of three sinusoidal waves, a combination of three square waves, a combination of three triangular waves and a combination of the three different above-mentioned waves. Obviously, the first source signal was continuous; the second was non-continuous and non-smooth; the third was continuous but non-smooth; and the final wave was non-continuous and non-smooth. First, their types and their corresponding numbers were known to us. Of 20 runs, our proposed SDDE approach could perfectly or almost perfectly decompose these four periodic source signals into separate waves. In contrast, empirical mode decomposition (EMD), the currently most widely-used data-driven decomposition tool, could not decompose these four periodic signals into separate waves. By nature, the outcomes of EMD, intrinsic mode functions, are combinations of amplitude-modulated modes and frequency-modulated modes; thus, EMD tends to view some periodic waves, single or composite, as its basis functions; and it also produces special non-periodic waves, generally transient and non-stationary, which sometimes are not easy to explain. In the second condition, one or three types of waves were known to us, but their corresponding numbers were unknown. Our proposed SDDE approach was still able to determine the main components even in the presence of 10 dB white noise.

Limited by a finite frequency resolution and sinusoidal basis functions, discrete Fourier transform (DFT) can decompose well only for signals with combinations of sinusoidal waves whose frequencies all match the frequency resolution. In contrast, our proposed SDDE approach can decompose well for combinations of various periodic waves. DFT and EMD well suit for roughly localizing the main components in order to provide much information about the source signal. With the help of DFT and EMD, our proposed SDDE approach can perform well in an efficient and effective way and detail more about how many main components indeed exist.

In the future, many works can be performed, including the use of source signals with more components, collecting various signal components—even complex components—into a collection of signal components as possible known types, and applying SDDE to some interesting source signals. In addition, we can adopt other advanced DE methods or other nature-inspired optimization algorithms, such as real-coded genetic algorithms or particle swarm optimization, to perform signal decomposition under newly developed objective functions. It can be expected that these objective functions may also be used as another set of benchmark functions, which are simple in theory but difficult in practice, to compare the performance among various optimization algorithms in the near future.

## Figures and Tables

**Table 1:** Performance of four source signals.

Runs	Sinusoidal	Square	Triangular	Composite
1	7.58E−02	0.00E+00	2.75E−01	6.80E−01
2	3.51E−32	0.00E+00	1.65E−01	9.10E−01
3	3.51E−32	0.00E+00	2.02E−03	0.00E+00
4	3.51E−32	0.00E+00	8.58E−02	1.69E−30
5	3.51E−32	0.00E+00	3.07E−02	5.09E−01
6	5.10E−22	0.00E+00	1.37E−03	1.24E+00
7	0.00E+00	0.00E+00	1.09E−01	8.09E−01
8	0.00E+00	0.00E+00	9.62E−01	1.12E−01
9	4.99E−01	0.00E+00	4.31E−31	4.84E−01
10	3.51E−32	0.00E+00	1.31E−03	1.40E+00
11	3.51E−32	0.00E+00	4.16E−01	5.09E−01
12	1.67E−01	0.00E+00	3.59E−01	6.31E−04
13	4.99E−01	0.00E+00	5.26E−03	7.97E−03
14	5.55E−32	0.00E+00	4.16E−01	1.95E−15
15	5.55E−32	0.00E+00	6.18E−01	1.13E+00
16	3.51E−32	0.00E+00	4.31E−01	6.80E−01
17	1.65E−30	0.00E+00	4.16E−01	7.78E−01
18	3.33E−02	0.00E+00	8.22E−02	1.24E+00
19	5.29E−03	0.00E+00	4.16E−01	1.49E+00
20	5.55E−32	0.00E+00	2.75E−01	8.34E−24
Mean	6.39E−02	0.00E+00	2.53E−01	5.99E−01
Std.	1.54E−01	0.00E+00	2.55E−01	5.19E−01

**Table 2:** Performance of four noised source signals (10 dB white noise) with known type and unknown number.

Runs	Sinusoidal	Square	Triangular	Composite
1	6.41E−01	1.56E+00	1.01E+00	7.54E−01
2	6.44E−01	1.55E+00	9.98E−01	7.65E−01
3	6.33E−01	1.52E+00	9.98E−01	7.81E−01
4	6.32E−01	1.54E+00	9.94E−01	7.56E−01
5	6.41E−01	1.55E+00	1.00E+00	8.77E−01
6	6.29E−01	1.57E+00	9.98E−01	7.70E−01
7	6.44E−01	1.56E+00	9.97E−01	8.67E−01
8	6.37E−01	1.53E+00	1.01E+00	7.44E−01
9	6.40E−01	1.54E+00	1.00E+00	9.34E−01
10	6.30E−01	1.53E+00	1.01E+00	9.30E−01
11	6.43E−01	1.52E+00	1.01E+00	7.67E−01
12	6.38E−01	1.53E+00	1.00E+00	7.62E−01
13	6.35E−01	1.55E+00	1.01E+00	7.54E−01
14	6.30E−01	1.54E+00	1.01E+00	8.23E−01
15	6.37E−01	1.55E+00	1.00E+00	8.74E−01
16	6.45E−01	1.56E+00	1.01E+00	7.72E−01
17	6.45E−01	1.55E+00	9.98E−01	7.67E−01
18	6.41E−01	1.54E+00	9.98E−01	9.11E−01
19	6.33E−01	1.54E+00	1.00E+00	7.50E−01
20	6.44E−01	1.55E+00	1.00E+00	8.46E−01
Mean	6.38E−01	1.54E+00	1.00E+00	8.10E−01
Std.	5.47E−03	1.24E−02	4.97E−03	6.58E−02
*P_s_*	7.09E+00	1.51E+01	1.02E+01	7.78E+00
*P_n_*	7.09E−01	1.51E+00	1.02E+00	7.78E−01
*P_r_*	6.58E−01	1.61E+00	1.03E+00	7.70E−01

**Table 3:** Performance of three noised source signals (10 dB white noise) with three known types and unknown number.

Runs	Sinusoidal	Square	Triangular
1	7.18E−01	1.32E+00	9.74E−01
2	7.18E−01	1.62E+00	1.00E+00
3	7.28E−01	1.47E+00	1.00E+00
4	7.53E−01	1.32E+00	1.00E+00
5	7.33E−01	1.43E+00	1.02E+00
6	7.28E−01	1.34E+00	1.02E+00
7	7.38E−01	1.32E+00	9.81E−01
8	7.20E−01	1.33E+00	9.97E−01
9	7.35E−01	1.39E+00	9.80E−01
10	7.36E−01	1.38E+00	9.80E−01
11	7.32E−01	1.39E+00	9.77E−01
12	7.31E−01	1.55E+00	9.81E−01
13	7.33E−01	1.30E+00	1.01E+00
14	6.96E−01	1.38E+00	9.63E−01
15	7.44E−01	1.61E+00	9.85E−01
16	7.71E−01	1.31E+00	1.01E+00
17	7.25E−01	1.47E+00	9.92E−01
18	7.39E−01	1.50E+00	9.93E−01
19	7.37E−01	1.50E+00	9.88E−01
20	7.28E−01	1.75E+00	1.00E+00
Mean	7.32E−01	1.43E+00	9.93E−01
Std.	1.48E−02	1.23E−01	1.58E−02
*P_s_*	7.09E+00	1.51E+01	1.02E+01
*P_n_*	7.09E−01	1.51E+00	1.02E+00
*P_r_*	7.55E−01	1.33E+00	1.03E+00

## Data Availability

Data sharing not applicable.
